# Deciphering the focuses and trends in skin regeneration research through bibliometric analyses

**DOI:** 10.3389/fmed.2022.947649

**Published:** 2022-07-22

**Authors:** Jian Zhou, Chen Dong, Qiuju Shu, Yang Chen, Qing Wang, Dandan Wang, Ge Ma

**Affiliations:** ^1^Savaid Stomatology School, Hangzhou Medical College, Hangzhou, China; ^2^Department of Prosthodontics, Xi’an Savaid Stomatology Hospital, Xi’an, China; ^3^Department of Plastic Surgery, Xijing Hospital, Fourth Military Medical University, Xi’an, China; ^4^Clinic of Dental Experts, Xi’an Savaid Stomatology Hospital, Xi’an, China; ^5^Department of Oral and Maxillofacial Surgery, Xi’an Daxing Hospital, Xi’an, China

**Keywords:** bibliometric analysis, skin regeneration research, tissue engineering, wound dressing, 3D bioprinting

## Abstract

Increasing attention to skin regeneration has rapidly broadened research on the topic. However, no bibliometric analysis of the field’s research trends has yet been conducted. In response to this research gap, this study analyzed the publication patterns and progress of skin regeneration research worldwide using a bibliometric analysis of 1,471 papers comprising 1,227 (83.4%) original articles and 244 (16.6%) reviews sourced from a Web of Science search. Publication distribution was analyzed by country/region, institution, journal, and author. The frequency of keywords was assessed to prepare a bibliometric map of the development trends in skin regeneration research. China and the United States were the most productive countries in the field: China had the greatest number of publications at 433 (29.4%) and the United States had the highest H-index ranking (59 with 15,373 citations or 31.9%). Author keywords were classified into four clusters: stem cell, biomaterial, tissue engineering, and wound dressing. “Stem cells,” “chitosan,” “tissue engineering,” and “wound dressings” were the most frequent keywords in each cluster; therefore, they reflected the field’s current focus areas. “Immunomodulation,” “aloe vera,” “extracellular vesicles,” “injectable hydrogel,” and “three-dimensional (3D) bioprinting” were relatively new keywords, indicating that biomaterials for skin regeneration and 3D bioprinting are promising research hotspots in the field. Moreover, clinical studies on new dressings and techniques to accelerate skin regeneration deserve more attention. By uncovering current and future research hotspots, this analysis offers insights that may be useful for both new and experienced scholars striving to expand research and innovation in the field of skin regeneration.

## Introduction

The skin is the largest organ and an essential part of the human body (1). By functioning harmoniously with other organs, the skin is the first body’s barrier against the damage from the external environment (2). “Skin regeneration” refers to the complete replacement of damaged skin with new skin (3). Impaired skin regeneration is a common outcome in patients with diabetes, pressure ulcers, and burns, making skin regeneration treatments necessary (4). However, global population growth has increased the demand for and costs of skin regeneration treatments (5). In light of this, skin regeneration has become an extensively researched subject (6).

Bibliometrics is a statistical method that involves quantitatively analyzing research papers on special topics via mathematical methods (7). Unlike traditional citation counts, a bibliometric approach considers the connections within the literature; notably, it identifies intellectual structures and emerging trends (8). Within the field of tissue regeneration, bibliometric analyses have been employed to estimate its research trends, including cardiac (9), neural (10), periodontal (11), cartilage (12), and bone regeneration (13). However, no bibliometric reports assessing the relevant scientific outputs and research trends of studies on skin regeneration have yet been published.

To fill this gap in the scholarly archive, this study sought to investigate the publication pattern and progress of skin regeneration research worldwide. Data were obtained from the Web of Science Core Collection (WoSCC). The publication distribution was systematically assessed by geography, institution, journal, and author. Furthermore, we assessed the frequency of keywords and conducted bibliometric mapping to uncover the development of skin regeneration research.

## Materials and methods

### Data sources and search strategies

WoSCC is a credible database for conducting bibliometric analyses across many publications (11, 12, 14). Therefore, we conducted a comprehensive online search of skin regeneration research from 1900 when WoSCC was launched, on 29 April 2022. To analyze the data, such as the number of publications annually and the number of articles published by country, institution, journal, and author, we downloaded plaintext versions of the articles. As this review used data obtained from a public database and did not involve human subjects, no ethical consent was required.

The search was only conducted on one day (29 April 2022) to prevent inconsistency caused by rapid database renewal. The search strategies were as follows: Topic = (“skin regeneration”) AND language = English. Original articles and reviews that had undergone a standard peer review and which appeared in Science Citation Index Expanded were eligible for inclusion. The two researchers (JZ and CD) performed a preliminary screening by reading the titles and abstracts of the skin regeneration literature presented by the search to exclude irrelevant papers. In the case of a disagreement, a third person (GM) assisted in resolving the discrepancy by reading the full text.

### Data collection

Two authors (JZ and CD) independently extracted data, including titles, keywords, publication dates, origin countries or regions, authors, institutions, journals, sums of citations, and H-indices, from correlative publications. The data from WoSCC were inputted into Microsoft Excel 2016 (Redmond, WA, United States), GraphPad Prism 7 (GraphPad Prism Software Inc., San Diego, CA, United States), and VOSviewer version 1.6.18 (Leiden University, Leiden, Netherlands) and were subsequently analyzed quantitatively and qualitatively. Meanwhile, the World Bank website was used to collect the latest information regarding the gross domestic product (GDP) of the countries/regions.

### Bibliometric analyses

Bibliometric analyses were conducted on the following aspects: growth trend of publications, publication countries/regions, journals, institutions, authors, keywords, and citations. We performed the calculations in the following order: (1) the contributions of countries/regions to global publications in terms of the number of publications produced, number of citations, H-index, GDP of the country/region, and country/region-wise co-authorship; (2) the publication distribution of different journals; (3) the frequency of different institutions and institution-wise co-authorship; (4) the authors with the most publications and the most cited papers, both in the research scope of skin regeneration and author-wise co-authorship; (5) clusters and emergence time of keywords; (6) and research progress in skin regeneration based on a constructed network of direct and co-citations of published papers.

We applied the web statistical tool in the Web of Science to analyze the characteristics of the included publications and then used VOSviewer to create a collaborative map based on countries/regions, institutions, and authors. The size of an item’s circle on the map was proportional to the number of its publications and the width of a line between two items was proportional to the magnitude of their collaboration. Items of the same color belonged to the same cluster, indicating that they cooperated closely in this field (9).

The main concepts in bibliometric analyses are briefly outlined below (15).

• Relative research interest (RRI) is determined by the total number of publications in all fields divided by the annual number of publications in one specific field; this eliminates bias from an increase in total publications.
• Average publication year (APY) is used to quantify the relative novelty of a keyword.
• Co-citation analysis involves tracking pairs of papers that are cited together in the source articles. When the same pairs of papers are co-cited by many authors, clusters of research begin to form. The co-cited papers in these clusters tend to share common themes.
• The total link strength of an item reflects the degree of cooperation with other items. The higher its value, the higher the level of cooperation (9).

## Results

### Growth trend of publications

**FIGURE 1:**
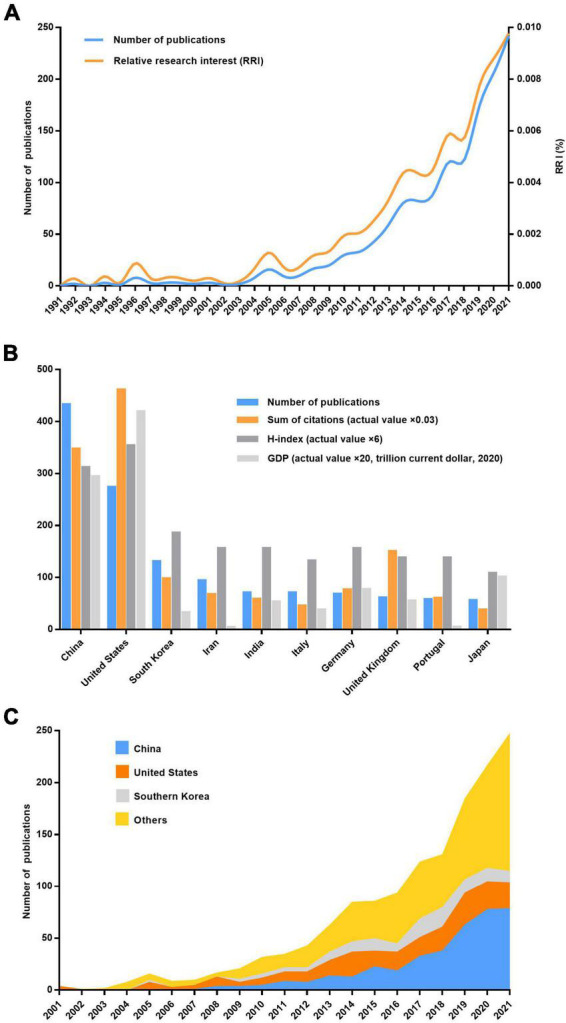
Contributions of different countries/regions to the field of skin regeneration. **(A)** Number of publications worldwide and the time course of relative research interest in skin regeneration; **(B)** number of publications, citation frequency (×0.03), H-index (×6), and GDP (×20, per trillion US dollars, 2020) of countries/regions (no less than five publications regarding skin regeneration); **(C)** number of publications from the top three and other countries per year (RRI, relative research interest).

A total of 1,471 publications, including 1,227 (83.4%) articles and 244 (16.6%) reviews, met our screening criteria. The number of articles on skin regeneration worldwide increased significantly over time, from 2 in 2000 to 242 in 2021 (Figure 1A). When considering the number of all-field publications, global interest in skin regeneration was measured in terms of the RRI, which was approximately 0.0003% before 2000 and 0.009% in 2021. In addition to the above publications, only one retracted publication was searched for incorrect data interpretation and inaccurate citation.

### Bibliometric analysis of countries/regions

**FIGURE 2:**
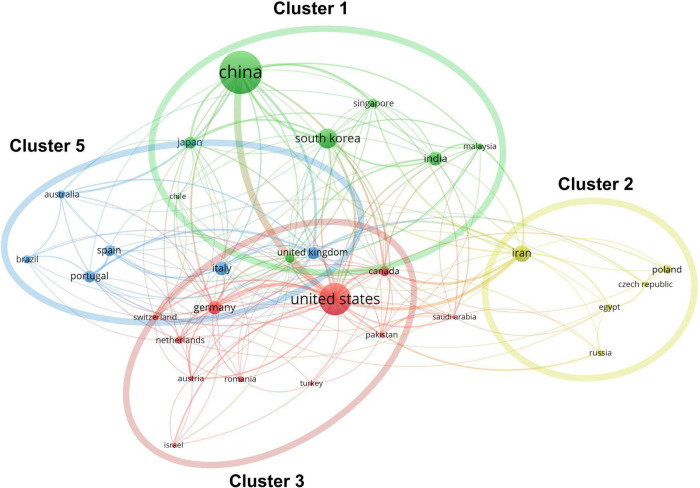
Co-authorship analysis of countries/regions divided into five clusters indicated with different colors. The large icon indicates countries/regions with high frequencies. Maximum number of countries per paper: 25; minimum number of countries per paper: 10; and minimum number of citations from one country: 10.

China ranked first for the number of publications at 433 (29.4%), followed by the United States at 274 (18.6%) and South Korea at 131 (8.9%; Figures 1B,C). However, regarding the H-index, the United States ranked first (59 with 15,373 citations or 31.9%), followed by China (52 with 11,575 citations or 24.0%) and South Korea (31 with 3,272 citations or 6.8%); these results are consistent with these countries’ 2020 GDP rankings. Moreover, four clusters were mapped by coauthors of countries/regions (Figure 2). Cluster 1 mainly included Western European countries/regions, such as England, France, and Netherlands. China, Iran, the United States, and Italy had the highest number of published papers in Clusters 1–4.

### Bibliometric analysis of journals, institutions, and department

**FIGURE 3:**
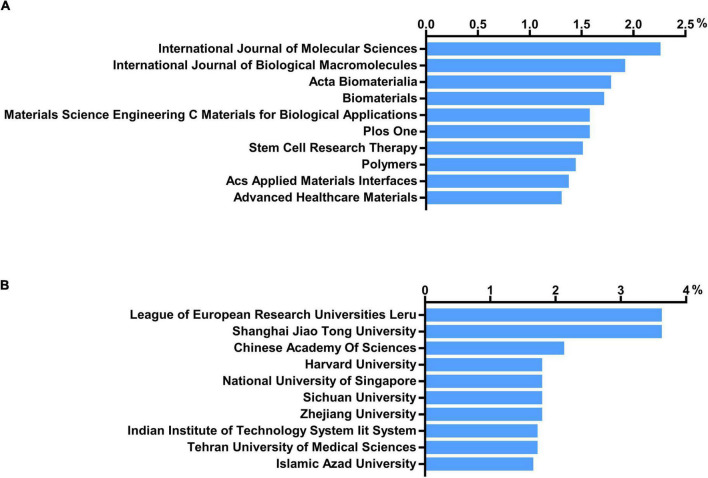
Number of institutions and journals focusing on skin regeneration. **(A)** Top 10 journals publishing research on skin regeneration; **(B)** top 10 institutes publishing research on skin regeneration.

**FIGURE 4:**
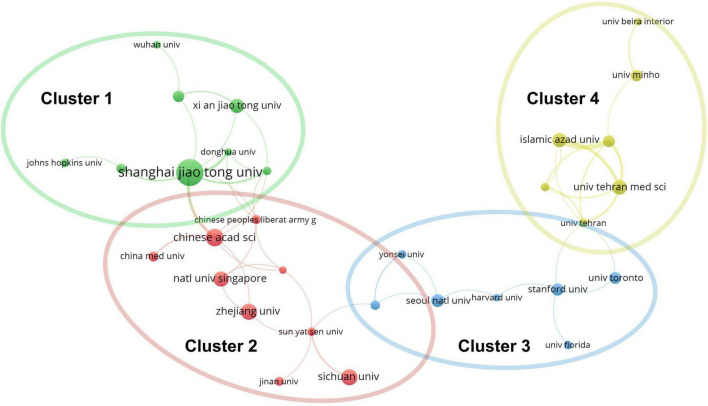
Co-authorship analysis of organizations divided into five clusters, indicated with different colors. The large icon indicates organizations with high frequencies. Maximum number of countries per paper: 25; minimum number of countries per paper: 10; and minimum number of citations from one country: 10.

Approximately one-sixth of the skin regeneration papers (240, 16.3%) were published in the top 10 journals ranked by number of publications. Specifically, the *International Journal of Molecular Sciences* (33 publications; 2.2%), the *International Journal of Biological Macromolecules* (28 publications; 1.9%), and *Acta Biomaterialia* (26 publications; 1.8%) were ranked first, second, and third, respectively. The top 10 journals with the most publications are listed in Figure 3A. With 53 papers (3.6%) each, the League of European Research Universities (LERU) and Shanghai Jiao Tong University have published more than any other institution in the world. The top 10 institutions with the most publications are listed in Figure 3B. An analysis of co-authorship between institutions showed five clusters (Figure 4). The institutions with the highest number of publications in Clusters 1–4 were Shanghai Jiao Tong University (50 publications), the Chinese Academy of Sciences (29 publications), Seoul National University (19 publications), and Tehran University of Medical Sciences (23 publications). Besides, in the top 100 most frequently cited publications, the department of biomedical engineering ranked first in the number of publications at 20, followed by materials science at 16 and biology at 10.

### Bibliometric analysis of authors

**FIGURE 5:**
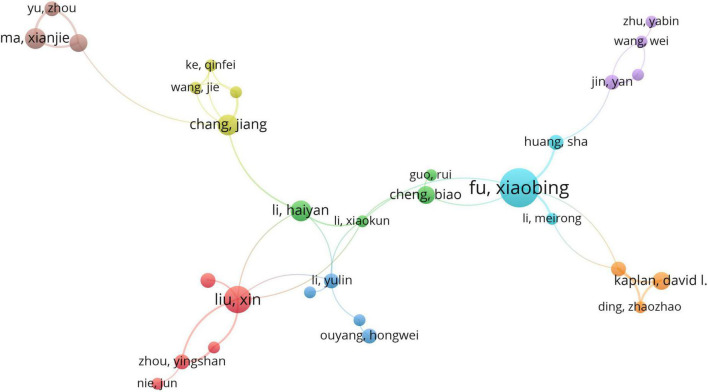
Co-authorship analysis of authors. The authors were divided into eight clusters, indicated with different colors. The large icon indicates authors with high frequencies. Maximum number of authors per paper: 25; minimum number authors per paper: 4; and minimum number of citations of one author: 4.

The top 10 authors wrote a total of 135 papers, accounting for 9.2% of all studies on skin regeneration. Li Qingfeng from Shanghai Jiao Tong University published 17 papers related to skin regeneration, thus ranking first in terms of the number of publications. Ramakrishna Seeram from the National University of Singapore and Fu Xiaobing from the Chinese People’s Liberation Army General Hospital ranked second and third with 15 and 14 papers, respectively. Furthermore, Lorna J. Gibson from the Massachusetts Institute of Technology had the highest citation frequency (Table 1 and Supplementary Table 1) (16–23). These highly cited articles are all basic science studies. The eight clusters of co-authorship among authors are illustrated in Figure 5; most show that Chinese authors cooperate relatively frequently.

**TABLE 1:** Top eight most frequently cited articles related to skin regeneration.

Title	Corresponding author	Journal	Publication year	Total citations (*n*)	Average citation per year (*n*)	Corresponding author’s country	Materials used	Cell/animal models
The effect of pore size on cell adhesion in collagen-GAG scaffolds	Gibson L. J	Biomaterials	2005	947	52.61	United States	Collagen and glycosaminoglycan	MC3T3-E1 mouse clonal osteogenic cells
Adhesive hemostatic conducting injectable composite hydrogels with sustained drug release and photothermal antibacterial activity to promote full-thickness skin regeneration during wound healing	Guo B. L.	Small	2019	448	112	China	Hyaluronic acid-graft-dopamine and reduced graphene oxide	Mouse full-thickness wounds model
Electrospun poly (lactic acid-co-glycolic acid) scaffolds for skin tissue engineering	Laurencin C. T.	Biomaterials	2008	415	27.67	United States	Electrospun poly (lactic acid-co-glycolic acid)	Human skin fibroblasts
Electrospun collagen/chitosan nanofibrous membrane as wound dressing	Chen J. K.	Colloids and Surfaces A: Physicochemical and Engineering Aspects	2008	369	24.6	China	Polycaprolactone–chitosan nanofibrous	Human keratinocyte and fibroblast cells
Electrospun water-soluble carboxyethyl chitosan/poly(vinyl alcohol) nanofibrous membrane as potential wound dressing for skin regeneration	Nie J.	Biomacromolecules	2008	353	23.53	China	Carboxyethyl chitosan/poly(vinyl alcohol) nanofibrous	Mouse fibroblasts (L929)
Bio-printed amniotic fluid-derived stem cells accelerate healing of large skin wounds	Soker S.	Stem Cells Translational Medicine	2012	350	31.82	United States	Amniotic fluid-derived stem cells	Nu/nu mice full-thickness wounds model
Dextran hydrogel scaffolds enhance angiogenic responses and promote complete skin regeneration during burn wound healing	Gerecht S.	Proceedings of the National Academy of Sciences of the United States of America	2011	310	25.83	United States	Dextran hydrogel	Mice third-degree burn wound model
Degradable conductive injectable hydrogels as novel antibacterial, anti-oxidant wound dressings for wound healing	Guo B. L.	Chemical Engineering Journal	2019	292	73	China	N-carboxyethyl chitosan and oxidized hyaluronic acid-graft-aniline tetramer	Mice full-thickness skin defect model

### Bibliometric analysis of keywords

**FIGURE 6:**
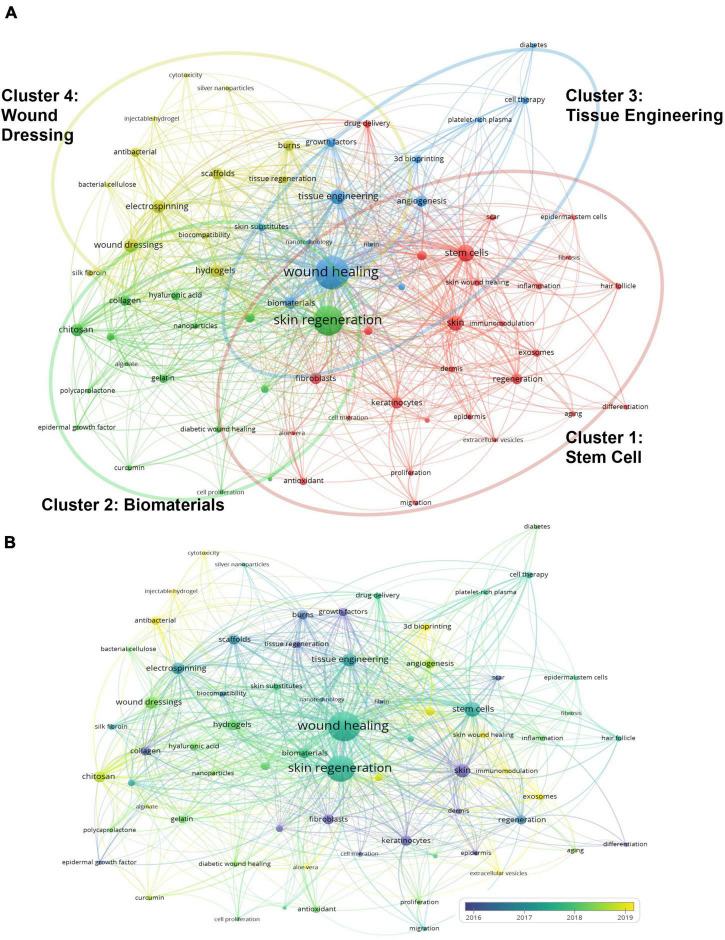
Analysis of keywords in publications on skin regeneration. **(A)** Map of the keywords concerning skin regeneration. The keywords were divided into three clusters, indicated with different colors. The large icon indicates keywords with high frequencies. **(B)** The keyword distribution is presented according to the average date of appearance, with blue representing an early appearance and yellow indicating a recent appearance. The smaller the distance between two keywords, the greater the frequency of their co-occurrence. Minimum number of occurrences of one keyword: 7.

A total of 3,054 author keywords were extracted from the publications using VOSviewer. As presented in Figure 6A, 69 keywords that occurred more than seven times were identified and classified into four clusters: stem cell (Cluster 1), biomaterial (Cluster 2), tissue engineering (Cluster 3), and wound dressing (Cluster 4). In Cluster 1, stem cells (106 times), skin (82 times), and keratinocytes (47 times) were the top three search terms. In Cluster 2, skin regeneration (329 times), chitosan (60 times), and collagen (38 times) were the most frequently searched terms. In Cluster 3, the top three keywords were wound healing (405 times), tissue engineering (86 times), and biomaterials (53 times). In Cluster 4, wound dressing (72 times), electrospinning (67 times), and hydrogels (60 times) appeared the most frequently.

As presented in Figure 6B, VOSviewer colored the keywords according to the date on which each word was published. Specifically, blue indicates that the word was published a relatively long time ago and yellow indicates that it was published recently (15). For example, early in the history of research on skin regeneration, the APY for “epidermis” (Cluster 1) was 2012.6. Meanwhile, “extracellular vesicles” (Cluster 1) is a relatively new keyword with an APY of 2020.3. “Immunomodulation,” “aloe vera,” and “extracellular vesicles”; “chitosan”, “alginate,” and “curcumin”; “three-dimensional (3D) bioprinting,” “angiogenesis,” and “diabetes”; and “injectable hydrogel,” “antibacterial,” and “wound dressing” were the top three most recent keywords in Clusters 1–4, respectively. The detailed results of the co-occurrence analysis of all keywords are presented in Supplementary Table 2.

### Bibliometric analysis of citations and co-citations

**FIGURE 7:**
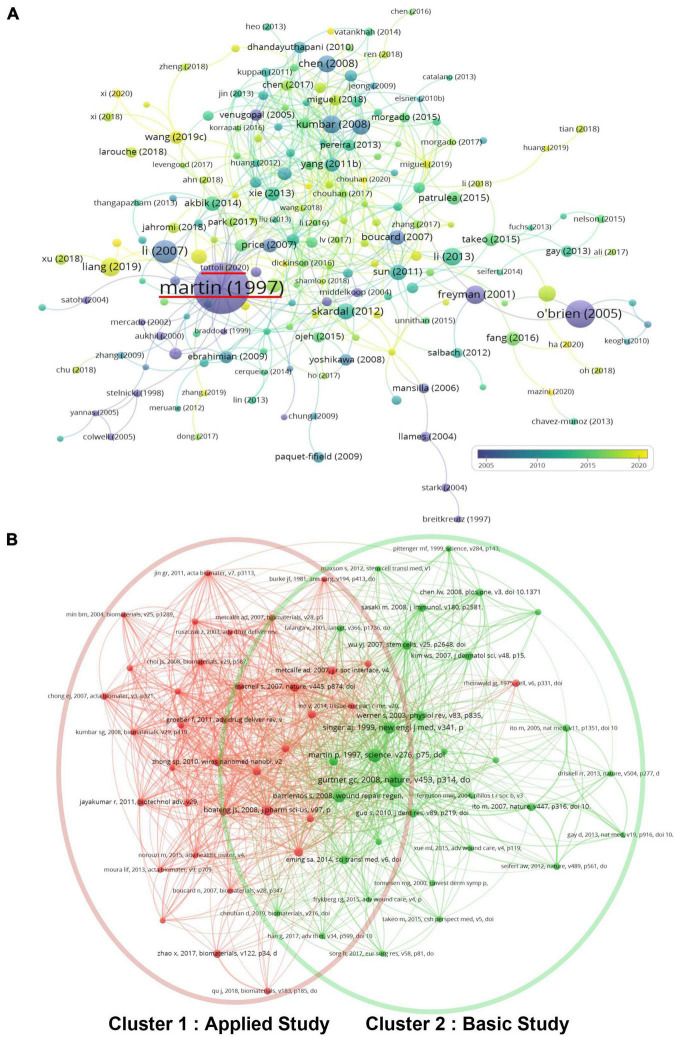
Citation and co-citation analysis of papers on skin regeneration. **(A)** Citation map; the distribution of publications is presented according to the average date of appearance, with blue representing an early appearance and yellow indicating a recent appearance; the red horizontal line indicates the most cited articles and the most cited articles among the latest articles; minimum number of times a paper was cited: 50. **(B)** Co-citation map; the publications were divided into three clusters in accordance with different colors. The large icon indicates publications with high frequencies; minimum number of citations of one document: 25.

All the articles related to skin regeneration considered in this study have been cited 48,171 times since 1990, with an average citation frequency of 32.7 times per paper and 1,459.7 times per year. To reveal whether these publications on skin regeneration were interlinked (i.e., whether they cited each other), VOSviewer was used to create a direct citation map of publications with over 50 citations (Figure 7A). In this map, Martin et al.’s (24) paper was most frequently cited (3,359 times) and Tottoli et al.’s (25) paper was the most cited (121 times) among recent publications. The bibliometric analysis results of co-citations (cited references) are shown in Figure 7B. Cluster 1, which had a similar keyword co-occurrence to Clusters 2–4, was named “applied study” while Cluster 2, which had a similar keyword co-occurrence to Cluster 1, was named “basic study.”

## Discussion

Young scholars and trained experts researching skin regeneration must find relevant scientific literature, clarify its past and current development, uncover important information, and identify active research frontiers and development trends in the field. This paper contributes to this work by summarizing these key concerns. More specifically, this study identified fundamental details of the skin regeneration field by conducting co-author analyses across journals, institutions, and authors.

The number of research publications on skin regeneration has increased rapidly over the last 30 years, indicating that skin regeneration is a research hotspot. Surveying this landscape, this study found that China is the most active country in skin regeneration research, particularly in terms of its total number of published articles. Accordingly, Chinese authors, such as Li Qingfeng and Fu Xiaobing, have been the most productive and active researchers on the topic. Notably, Li Qingfeng, the most productive author in the field, studies mechanical induced skin regeneration. Recently, his team identified EZH2 and CDH1 as therapeutic targets for skin regeneration after mechanical loading (26, 27). However, in terms of the number of total citations and H-index, the United States is the most influential country in the field of skin regeneration—this may be related to the United States’ early start and significant financial investment in the field. Notably, China and the United States frequently cooperate in skin regeneration research, as illustrated by the co-occurrence network of countries/regions. Generally, this study found that European and Asian authors are increasingly contributing to research on skin regeneration and that the influence of authors from the United States is extremely significant. Meanwhile, the *International Journal of Molecular Sciences*, an international, peer-reviewed, open access journal providing an advanced forum for all aspects of molecular research, published the largest number of studies on skin regeneration. Regarding institutions, the LERU has published the largest number of articles in the field.

### Research focuses

The co-occurrence analysis of author keywords yielded the following four clusters (Clusters 1–4) of keywords based on the bibliographic map. The more frequently the keywords occur, the more noteworthy the related topics. In addition to keywords representing skin regeneration itself, such as “skin regeneration” and “wound healing,” the keywords revealed the following focus areas.

“Stem cells” was the most frequent keyword in Cluster 1. When the skin barrier is breached during wounding, re-establishing tissue integrity and function requires considerable coordination between various cell types, signaling factors, and matrix interactions (28). In this process, tissue-resident stem cells play an important role in self-renewing and maintaining their population during homeostasis (28). The stem cells must also cooperate with other cell types, including fibroblasts and immune cells, to ensure efficient and harmonious skin regeneration during wound healing (28). An exogenous addition of stem cells to skin injury lesions increases cell proliferation and neovascularization while reducing inflammation (29). Moreover, stem cell-based therapeutic strategies have shown considerable potential for improving the rate and quality of wound healing and skin regeneration (30).

“Chitosan” was the most frequently seen in Cluster 2. Chitosan is one of the most frequently studied biomaterials. It is formed by N-acetyl-D-glucosamine monomers with β-1,4-glycosidic bonds and is acquired by deacetylating the chitin extracted from crustacean shells. Studies have reported many benefits of chitosan and its derivatives in skin regeneration, including its desirable pharmacological value (due to its antibacterial, anti-inflammatory, and hemostatic properties) (31, 32); superior biocompatibility and biodegradability (33); good water absorption and retention properties; and the amino (−NH2) and hydroxyl (−OH) groups in its molecular chains, which enable it to graft to other groups and chemical components to enhance particular biological functions (34). When combined with other materials—namely, biological macromolecules or bioactive factors—chitosan is more effective in promoting skin regeneration, as seen with chitosan microneedle array patches and chitosan-based hydrogels with nanotechnologically modified curcumin and epidermal growth factors (35, 36). Moreover, the treatment effect of a chitosan dressing has been tested in many clinical trials, which reveal that chitosan acts as an effective antimicrobial and procoagulant agent, provides beneficial microbiota, facilitates wound re-epithelialization, and reduces patients’ pain levels (37–39).

“Tissue engineering” was the most frequent keyword in Cluster 3. Over the past few years, tissue engineering has enormously contributed to the progress of skin regeneration and wound healing (40). Through the use of biomaterials, bioactive molecules, cells, and their combinations, skin tissue engineering develops engineered scaffolds that can assist skin reconstruction (41). Skin can be replaced or modeled with tissue-engineered constructs that mimic native physiological characteristics (42). In clinical applications, engineered skin substitutes are effective in accelerating wound healing in cases of extensive burns, venous leg ulcers, and diabetic foot ulcers (43–45). Nevertheless, there is a need to redesign the currently available alternatives to make them more user-friendly, affordable, and viable (30). Other trending topics in tissue engineering include functional artificial skin grafts for nerve reconstruction, pigmentation, and skin appendages (e.g., hair follicles and sweat glands) (46).

“Wound dressings” was the most frequent keyword that appeared in Cluster 4. For several millennia, primary wound dressings, such as plasters, have served as physical barriers to protect wounds from the external environment (47). Over time, newer wound dressing materials have been developed to provide appropriate care for wounds and ensure optimal healing (48). Apart from chitosan, as previously described, collagen (49), alginate (50), cellulose (51), gelatin (52), hyaluronic acid (53), silk (54), and foam dressings (55) have been extensively studied in the field of skin wound dressings. In recent years, novel wound dressings have served as not only physical and chemical barriers, but also real-time monitors of the wound environment by allowing the growth factor and cellular delivery to be monitored and act as antimicrobial barriers (56–58).

### Research trends

This study found that “immunomodulation” (Cluster 1), “aloe vera” (Cluster 1), “extracellular vesicles” (Cluster 1), “injectable hydrogel” (Cluster 4), and “3D bioprinting” (Cluster 3) are the newest keywords representing trends in skin regeneration research. These keywords suggest that emerging research trends are concerned with biomaterials that can facilitate skin regeneration. Below, the insights on biomaterials and skin regeneration uncovered by this study are presented.

First, bioactive components extracted from aloe vera have complex constituents and various pharmacological properties (59), such as antioxidant, anti-inflammatory, immunomodulatory, antimicrobial, antiviral, antidiabetic, hepatoprotective, anticancer, skin-protective, and wound-healing properties. These properties have been attributed to the presence of many active compounds, including anthraquinones, anthrones, chromones, flavonoids, amino acids, lipids, carbohydrates, vitamins, and minerals (60). When aloe vera is applied externally, it accelerates the regeneration of damaged skin (61, 62). Its healing property stems from a compound called glucomannan, which affects the fibroblast growth factor and stimulates the activity and proliferation of these cells, consequently improving collagen production and secretion (63). Thus, recent functional wound dressings contain aloe vera extracts as bioactive agents in combination with other scaffold materials (64–66).

Second, extracellular vesicles or exosomes have recently gained tremendous attention in the field of skin regeneration. These nanosized extracellular particles can break cellular boundaries and facilitate intracellular signal delivery during tissue regeneration (67). By conveying functional cargos (e.g., growth factor, cytokine, and miRNA) to target cells, extracellular vesicles not only participate in normal physiological processes, such as hemostasis, inflammation, proliferation, and remodeling, but also serve as a new style of wound treatment, particularly when derived from stem cells (68, 69).

Third, hydrogels were designed to gel at body temperature for injectable delivery for *in situ* forming (70). Compared with the traditional hydrogel formed outside of a patient or implanted using invasive surgical techniques, the injectable counterparts have several advantages, such as low cost, convenience, ability to deliver therapeutic payloads with minimal invasiveness, and ability to fill complex tissue defects (71, 72). Numerous chemical and material processing techniques have been utilized to produce injectable hydrogels, which are broadly categorized as covalent and non-covalent hydrogels (72). These materials can be injected as viscous liquids and subsequently solidified through variations in their local microenvironment (temperature, pH, and ion concentration), the application of an external stimulus (light), or affinity-based self-organization (in the case of peptides and other physically associating functional moieties) (73–78). The composite materials, formed by combining injectable hydrogels with other materials and bioactive components or active cells, exhibit great potential applications for skin regeneration (79–81).

Additionally, because immunomodulation plays a crucial role in skin regeneration (82, 83), specific cell-laden immunostimulating biomaterials may potentially be applicable in skin tissue restoration (84). Chen et al. (85) found that a combination of cryogel/hydrogel biomaterials and acupuncture can promote diabetic skin wound healing through immunomodulation. Meanwhile, Saleh et al. (86) used adhesive hydrogels loaded with miRNA-laden nanoparticles to promote wound healing caused by the polarization of macrophages to the M2 phenotype.

Moreover, 3D bioprinting can bridge the divergence between artificial tissue constructs and natural tissues; specifically, computer-aided design techniques can stack cell-laden materials layer by layer into 3D structures (87, 88). 3D bioprinting has several advantages for rapidly creating prototypes of customized structures, delivering cell-laden materials with high precision in space, and tissue engineering in a highly controllable microenvironment (89). Scholars have developed various bioprinting strategies on the basis of their fundamental working principles for fabricating functional tissue constructs, such as inkjet-based bioprinting, laser-assisted bioprinting, pressure-assisted (extrusion) bioprinting, acoustic bioprinting, stereolithography-based bioprinting, and magnetic bioprinting (90, 91). Through these strategies, 3D-printed skin possesses enormous potential as grafts for wound healing, burned skin replacement, and *in vitro* human skin modeling for product and drug testing (92–94). Notably, 3D bioprinting depends heavily on bioink for the development of functional organs or tissues. Recent studies have focused on bioinks used in 3D bioprinting, such as gelatin methacryloyl, collagen, and extracellular matrix collagen-based hydrogel (95).

Despite the obvious advantages of biomaterials and 3D bioprinting, most studies on these topics are experimental or preclinical. While no strict randomized controlled clinical trials are listed on WoSCC, ClinicalTrials.gov suggests that clinical trials in the field are currently underway. Moving forward, attention should be paid to clinical studies on how biomaterials and 3D bioprinting may be applied to accelerate skin regeneration.

This study has two main limitations. First, given the large number of relevant terms, it is difficult to guarantee the inclusion of all relevant articles and the exclusion of all articles that are largely irrelevant to the bibliometric analysis. Second, some of the latest publications were not emphasized in the study due to the common limitations of bibliometric analysis: if studies appear for only a short time and are insufficient in terms of number and frequency of cited literature, then a bibliometric analysis will not identify them. To be sure, this does not mean that recent literature is unimportant, but that more time is necessary for testing.

## Conclusion

In conclusion, this study deciphered the progress of skin regeneration research using a bibliometric analysis. It found that the number of articles on skin regeneration worldwide has significantly increased over time. China and the United States have been the most productive in the field. Going forward, the application of biomaterials that facilitate skin regeneration and 3D bioprinting are promising research hotspots; moreover, clinical studies on new dressings and techniques to accelerate skin regeneration deserve attention.

## Data availability statement

The original contributions presented in the study are included in the article/Supplementary Material, further inquiries can be directed to the corresponding author.

## Author contributions

JZ and CD conceptualized, designed, and conducted the study, acquired and analyzed the data, and wrote the manuscript. QS, YC, QW, and DW conducted the study and edited the manuscript. GM designed the study and wrote and edited the manuscript. All authors contributed to the article and approved the submitted version.
