# Supplementary material for: Deciphering the focuses and trends in skin regeneration research through bibliometric analyses
Source: Front Med (Lausanne). 2022 Jul 22;9:947649. doi: 10.3389/fmed.2022.947649 (PMC9355679; doi:10.3389/fmed.2022.947649)
Supplement: Supplementary file 2 [file Table_2.docx]

| **Supplemental Table 2.** The consequences of the co-occurrence analysis of 69 author keywords with at least 7 occurrence times | | | | | |
| --- | --- | --- | --- | --- | --- |
| Keywords | Cluster | Occurrences | Links | Average publication years (APY) | Average citations |
| Stem cells | 1 | 106 | 50 | 2017.5 | 30.7 |
| Skin | 1 | 82 | 44 | 2015.9 | 34.9 |
| Keratinocytes | 1 | 47 | 30 | 2015.12 | 15.4 |
| Fibroblasts | 1 | 45 | 31 | 2016.1 | 16.1 |
| Regeneration | 1 | 44 | 35 | 2016.9 | 17.2 |
| Regenerative medicine | 1 | 35 | 32 | 2018.11 | 13.4 |
| Chronic wounds | 1 | 24 | 33 | 2018.11 | 64.2 |
| Exosomes | 1 | 23 | 21 | 2019.12 | 33.4 |
| Antioxidant | 1 | 21 | 17 | 2018.3 | 33.4 |
| Drug delivery | 1 | 20 | 26 | 2017.10 | 52.4 |
| Skin wound healing | 1 | 19 | 17 | 2019.2 | 18.7 |
| Scar | 1 | 16 | 20 | 2016.4 | 23.2 |
| Hair follicle | 1 | 15 | 12 | 2017.5 | 22.1 |
| Inflammation | 1 | 15 | 22 | 2018.3 | 34.9 |
| Dermis | 1 | 14 | 19 | 2013.2 | 38.7 |
| Epidermis | 1 | 13 | 13 | 2012.6 | 26.6 |
| Proliferation | 1 | 13 | 11 | 2018.4 | 10.5 |
| Immunomodulation | 1 | 12 | 20 | 2020.4 | 35.3 |
| Epidermal stem cells | 1 | 11 | 13 | 2017.10 | 11.0 |
| Adipose-derived stem cells | 1 | 10 | 14 | 2018.1 | 17.3 |
| Migration | 1 | 10 | 10 | 2017.5 | 21.5 |
| Aging | 1 | 9 | 10 | 2018.5 | 16.2 |
| Differentiation | 1 | 9 | 6 | 2015.8 | 20.9 |
| Cell migration | 1 | 8 | 8 | 2016.1 | 20.9 |
| Extracellular vesicles | 1 | 8 | 14 | 2020.3 | 19.3 |
| Aloe vera | 1 | 7 | 14 | 2020.4 | 10.3 |
| Fibrosis | 1 | 7 | 10 | 2018.1 | 29.7 |
| Skin regeneration | 2 | 329 | 64 | 2017.6 | 23.5 |
| Chitosan | 2 | 60 | 32 | 2018.9 | 30.8 |
| Collagen | 2 | 38 | 29 | 2016.3 | 51.3 |
| Skin tissue engineering | 2 | 34 | 27 | 2018.2 | 28.8 |
| Gelatin | 2 | 31 | 26 | 2018.3 | 26.2 |
| Hyaluronic acid | 2 | 29 | 23 | 2018.3 | 27.0 |
| Nanofibers | 2 | 23 | 24 | 2017.3 | 46.3 |
| Extracellular matrix | 2 | 22 | 20 | 2013.12 | 30.3 |
| Diabetic wound healing | 2 | 18 | 18 | 2018.4 | 51.4 |
| Nanoparticles | 2 | 13 | 20 | 2018.5 | 50.8 |
| Curcumin | 2 | 12 | 12 | 2018.8 | 45.3 |
| Polycaprolactone | 2 | 10 | 15 | 2018.4 | 19.4 |
| Epidermal growth factor | 2 | 9 | 12 | 2016.5 | 39.4 |
| Alginate | 2 | 8 | 13 | 2018.9 | 14.9 |
| Cell proliferation | 2 | 7 | 10 | 2018.1 | 13.1 |
| Conditioned medium | 2 | 7 | 9 | 2017.11 | 15.4 |
| Wound healing | 3 | 405 | 67 | 2017.7 | 32.3 |
| Tissue engineering | 3 | 86 | 41 | 2017.2 | 41.2 |
| Biomaterials | 3 | 53 | 38 | 2017.10 | 27.5 |
| Angiogenesis | 3 | 49 | 39 | 2018.7 | 32.8 |
| Growth factors | 3 | 32 | 28 | 2015.10 | 53.3 |
| 3D bioprinting | 3 | 26 | 21 | 2020.1 | 27.5 |
| Skin substitutes | 3 | 21 | 23 | 2017.11 | 51.6 |
| Cell therapy | 3 | 20 | 13 | 2017.5 | 42.2 |
| Vascularization | 3 | 17 | 18 | 2017.8 | 27.5 |
| Platelet-rich plasma | 3 | 12 | 13 | 2017.10 | 17.3 |
| Diabetes | 3 | 10 | 6 | 2018.2 | 11.9 |
| Fibrin | 3 | 7 | 11 | 2016.4 | 22.1 |
| Nanotechnology | 3 | 7 | 17 | 2016.10 | 51.0 |
| Wound dressings | 4 | 72 | 37 | 2018.4 | 44.3 |
| Electrospinning | 4 | 67 | 38 | 2016.12 | 47.0 |
| Hydrogels | 4 | 60 | 39 | 2018.2 | 31.2 |
| Scaffolds | 4 | 52 | 36 | 2016.11 | 43.7 |
| Burns | 4 | 42 | 35 | 2016.6 | 23.9 |
| Antibacterial | 4 | 32 | 21 | 2019.3 | 28.4 |
| Tissue regeneration | 4 | 22 | 25 | 2015.11 | 84.6 |
| Biocompatibility | 4 | 16 | 17 | 2016.10 | 64.8 |
| Silk fibroin | 4 | 15 | 18 | 2017.3 | 31.3 |
| Bacterial cellulose | 4 | 10 | 13 | 2018.2 | 40.4 |
| Cytotoxicity | 4 | 7 | 8 | 2018.11 | 7.7 |
| Injectable hydrogel | 4 | 7 | 8 | 2020.3 | 61.7 |
| Silver nanoparticles | 4 | 7 | 12 | 2017.3 | 12.0 |
